# Why are population growth rate estimates of past and present hunter–gatherers so different?

**DOI:** 10.1098/rstb.2019.0708

**Published:** 2020-11-30

**Authors:** Miikka Tallavaara, Erlend Kirkeng Jørgensen

**Affiliations:** 1 Department of Geosciences and Geography, University of Helsinki, PO Box 64, 00014 Helsinki, Finland; 2 Department of Archaeology, History, Religious Studies and Theology, UiT - The Arctic University of Norway, PO Box 6050 Langnes, 9037 Tromsø, Norway

**Keywords:** hunter–gatherers, population growth rates, human population dynamics, archaeological population proxies, forager population paradox

## Abstract

Hunter–gatherer population growth rate estimates extracted from archaeological proxies and ethnographic data show remarkable differences, as archaeological estimates are orders of magnitude smaller than ethnographic and historical estimates. This could imply that prehistoric hunter–gatherers were demographically different from recent hunter–gatherers. However, we show that the resolution of archaeological human population proxies is not sufficiently high to detect actual population dynamics and growth rates that can be observed in the historical and ethnographic data. We argue that archaeological and ethnographic population growth rates measure different things; therefore, they are not directly comparable. While ethnographic growth rate estimates of hunter–gatherer populations are directly linked to underlying demographic parameters, archaeological estimates track changes in the long-term mean population size, which reflects changes in the environmental productivity that provide the ultimate constraint for forager population growth. We further argue that because of this constraining effect, hunter–gatherer populations cannot exhibit long-term growth independently of increasing environmental productivity.

This article is part of the theme issue ‘Cross-disciplinary approaches to prehistoric demography’.

## 1. Introduction

Population growth rate is one of the key demographic parameters. It has been argued that population growth rate can be taken as the unifying variable linking population dynamics to evolutionary processes [1] as well as the various aspects of population ecology such as density dependence, resource and interference competition and the effects of environmental stress [2]. Furthermore, an organism's niche can be defined as the set of environmental conditions where the population growth rate is greater than zero [2].

With increasingly sophisticated methods of analysing prehistoric demography, archaeologists have also started to measure growth rates of past human populations from archaeological proxies such as summed probability distributions (SPDs) of archaeological radiocarbon dates. It is now argued that population growth rate estimation offers great potential for population ecological and biodemographic research in archaeology [3]. For example, analysis of the growth rates in the archaeological radiocarbon date record has made it possible to compare expansion dynamics of human populations in South America to a general density-dependence model for invasive species [4]. Similarly, growth rate analysis has allowed parametrizing demographic models to calculate the total number of births per woman required to maintain the inferred annual growth rate among hunter–gatherers in prehistoric Wyoming and Colorado [5]. Yet, to what extent are current methods for quantifying human population growth in the past accurate, and what do they actually measure? Providing an answer to these questions is pertinent if archaeologists want to make a lasting contribution to human demography and population ecology.

Making inferences about growth rates of prehistoric populations is far from straightforward. For example, anthropologists have pointed out what appears to be a contradiction between relatively high hunter–gatherer population growth rates observed in the ethnographic record and the inferred long-term stationarity of the human population over much of our evolutionary history [6–8]: the annual population growth rates of 1–3% observed among ethnographic hunter–gatherers implies that a population of 100 foragers would have grown to billions in less than 2000 years, which obviously has never happened. This contradiction has been called the *forager population paradox* [9]. A number of authors have argued that the paradox is explicable only if the early human population history was characterized by rapid growth periods followed by crashes [6,8,10]. By providing the best window to past long-term demographic processes, archaeological population proxies could resolve this paradox.

Here, we evaluate the correspondence between population growth rates of prehistoric hunter–gatherers and those observed in more recent, ethnographic and historical records. This is done by comparing archaeological and ethnographic population growth rate estimates and temporal dynamics of hunter–gatherer populations. We show that the growth rates of prehistoric hunter–gatherers appear to be much lower than ethnographic growth rates. This could imply that prehistoric hunter–gatherers indeed were different from ethnographically and historically documented hunter–gatherers in terms of their demographic properties. However, using simulated data, we show that the resolution of archaeological human population proxies is not sufficiently high to detect the fine-grained population dynamics and, therefore, growth observed in the historical and ethnographic data. Consequently, we argue that archaeological and ethnographic population growth rates essentially measure different things, and therefore, they are not directly comparable. We further argue that the relationship between archaeological growth rates and demographic parameters, such as birth and death rates of the underlying prehistoric population, is not sufficiently established. Therefore, the current paradigm for archaeological growth rate estimation may miss the actual growth rate and population dynamics of prehistoric hunter–gatherers.

## 2. Material and methods

### (a) Archaeological data

To compare archaeological and ethnographic hunter–gatherer population growth rate estimates, we compiled such estimates from the existing literature. In our sample, archaeological growth rates are all calculated from the temporal distributions of radiocarbon dates—mostly SPDs—and derived from recent studies of prehistoric population growth [4,5,11–14]. The method of calculating the growth rate can influence results and affect comparison between different estimates, as for example, the average annual growth rates and mean annualized growth rates may not be entirely comparable [3]. Furthermore, taphonomic correction of temporal distribution of radiocarbon dates [15] also influences the growth rate calculated from SPDs [5]. When population growth is measured over thousands of years, the growth rate estimate would be lower if the taphonomic correction is applied to the data than without the correction. Despite these complications, we took published growth rate estimates as given and assume that the estimates are comparable.

### (b) Ethnographic and historical data

Our ethnographic sample consists of classical examples of hunter–gatherer populations from the tropics [6,9,16–18], complemented by historical demographic data from North European Sámi people [19–21]. Like archaeological population growth rate estimates, ethnographic estimates have been measured with different methods using census data: change in population size through time [6,17,18]; crude birth, death and migration rates [17]; life table analysis complemented by simulation [16]; and through combination of different methods [9]. These methods can produce slightly different results and, strictly speaking, they do not measure exactly the same demographic parameters.

Historical Sámi data make it possible to track temporal population dynamics of hunter–gatherers. We use tax records data from Guovdageaidnu (Kautokeino) and Ávjovárri (Karasjok) Sámi communities in northern Norway [21] and from the historical Kemi Lappi region in northern Finland that covers the Sámi communities of Anár (Inari), Kittilä, Sodankylä, Sompio, Kemikylä, Kuolajärvi, Maanselkä and Kitkajärvi [19,20]. Tax record data cover the periods AD 1553–1752 in northern Norway and 1555–1701 in northern Finland. Both datasets have gaps, the longest being between years 1621 and 1637. During this period, tax collecting privileges were switched from the Swedish crown to so-called *birkarl* traders, and therefore, no tax records were preserved [19,20].

For over 200 years, large-scale reindeer herding has been a key component of the inland Sámi economy. Before that, Sámi people were hunter–gatherers who used domesticated reindeer mainly for transportation and hunting decoys [22–24]. In 1605, the number of reindeer per owner in northern Norway and Finland was *ca* 10 at best and usually much less [24]. By the end of the seventeenth century, large-scale reindeer herding started to gain more foothold in northern Norway [22–24], although an earlier date for reindeer pastoralism has also been suggested [25]. In the Kemi Lappi region in Finland, this economic change occurred much later during the eighteenth and nineteenth centuries [20]. At the end of the seventeenth century, Finnish peasant settlers started a northward expansion in the Kemi Lappi region in Finland, and by the end of the eighteenth century, most local Sámi were assimilated into the agricultural Finnish population everywhere in the Kemi Lappi region, except in Anár [20].

In the study area, detailed demographic records began in the mid-eighteenth century when the economic shift from foraging to reindeer herding or farming was already underway. However, tax records extend much further back in time and provide rare data on longer-term population hunter–gatherer dynamics. Although tax records do not directly measure population size, the number of taxpayers can be used as a proxy for population size [19–21,26]. Basically, tax records count the number of adult males. Tax records also include the number of adult males that have not been able to pay taxes, e.g. because of poverty, but this inclusion is not systematic. The number of taxpayers can be transferred to population sizes by multiplying the number with the assumed family size [21,26]. For the Guovdageaidnu and Ávjovárri data, this transfer has been done [21]. For the Kemi Lappi data, we rely on the raw number of taxpayers and assume that it reflects population size [19,20].

For Sámi time-series data, population growth rate *r* is calculated using the following formula:

$$r=\frac{\text{ln}(N_{t}/N_{0})}{t}$$

where *N*_0_ is the size of the population at the start of growth, and *N_t_* is the size of the population at time *t* [27].

### (c) Simulated hunter–gatherer population dynamics

Historical records on hunter–gatherer population dynamics that cover more than just a few decades are hard to obtain. However, several studies have attempted to simulate longer-term hunter–gatherer population dynamics [28–31]. In the simulations of Wintehalder *et al*., a foraging population grows or declines as a function of the net marginal product of hunting and gathering, which is affected by food abundance that responds logistically to their exploitation [28,29]. In these simulations, changes from stable equilibrium to damped and stable population cycles occurred with modest adjustments of parameters.

Using different population and foraging models, Belovsky's simulation suggested that damped and, especially, stable cycles are common types of dynamics among hunter–gatherer populations [30]. These cycles are the result of the interplay between hunter–gatherer population density and the food abundance and composition of their diet. In Belovsky's simulation, hunter–gatherer population growth is based on nutritional intake and expenditure (survival and reproduction) in different environmental productivity regimes and is not informed by ethnographic growth rate estimates [30].

The potential commonality of population fluctuations is further demonstrated by Hamilton and Walker [31]. Unlike the other two simulation approaches, Hamilton and Walker do not consider density-dependent predator-prey dynamics but the effects of demographic and environmental stochasticity and periodic catastrophic events on hunter–gatherer population dynamics [31]. Their simulation yields highly fluctuating populations susceptible to local extinction events every few hundred years.

Despite differences in the underlying assumptions and models, a common characteristic among recent modelling studies is that they suggest hunter–gatherer populations gravitating towards oscillating dynamics, where periods of rapid growth are followed by periods of rapid decline. Here, we use Belovsky's simulation [30] as an example of theoretically expected hunter–gatherer dynamics. Because Belovsky's simulation is not informed by ethnographic population growth rates, it is possible to compare the simulated growth rates and temporal patterns to ethnographic and historical growth rates as well as to historical temporal dynamics. Furthermore, Belovsky's simulation provides interesting insight into the impacts of environmental productivity on hunter–gatherer population dynamics. Therefore, we use Belovsky's simulation also as a template for simulated archaeological population proxies.

### (d) Simulation of archaeological population proxies

SPDs are currently the most commonly used archaeological population proxy [4,5,12,13,32–34]. They are based on the idea that in a given study area, the number of radiocarbon-dated events falling into a given period is related to the number of people that lived in that period in that area. Therefore, temporal changes in the number of radiocarbon dates reflect relative changes in population size through time. To evaluate how well SPDs capture population dynamics suggested by simulation studies and historical data, we created simulated SPDs under the assumption that the underlying population dynamics would follow the pattern suggested by Belovsky's simulation [30]. We combined Belovsky's simulations in different environmental productivity regimes as long time series (figure 2) and sampled 5000 calendar dates between 10 000 and 5881 years ago using the simulated pattern as a weight in the sampling process. This set-up assumes that there occurred three step-like changes in the environmental productivity during the simulation age range. In addition, we created a scenario where environmental productivity remained constant. Following the process developed in Shennan *et al.* [34], we then converted sampled calendar ages to radiocarbon dates, added random error, recalibrated the radiocarbon dates and created SPDs. Error terms were randomly sampled from truncated normal distribution (*μ* = 50, *σ* = 15, *a* = 20, *b* = 80). We repeated the whole process several times to evaluate the sample variation. Finally, we used wavelet coherence analysis [35–37] to address the similarity between SPDs and the underlying population pattern.

All simulation and data analyses were performed in R [38] using packages rcarbon [39], truncnorm [40] and WaveletComp [37]. The R-script and data are available at the Zenodo data repository.

## 3. Results

**Figure 1.**
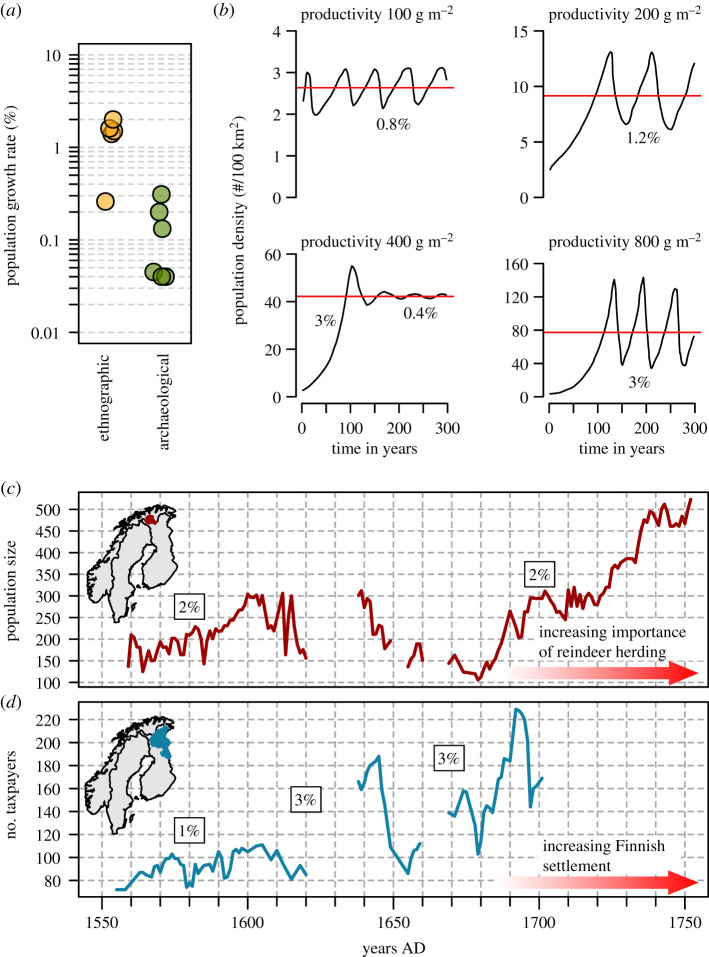
Archaeological hunter–gatherer population growth rates as compared to ethnographic, simulated and historical estimates. (*a*) Ethnographic and archaeological estimates of annual change in population size (table 1). (*b*) Belovsky's simulation of hunter–gatherer population dynamics in environments with different productivity [30]. Horizontal lines indicate mean population density. (*c*) Sámi population size in Guovdageaidnu and Ávjovárri communities in northern Norway [21]. (*d*) Number of taxpayers in the Kemi Lappi region in northern Finland [19,20]. This is assumed to reflect the Sámi population size in the region. (*b–d*) Show annual population growth rates (%) during growth periods.

Figure 1*a* and table 1 show that there are clear differences between ethnographic and archaeological population growth rate estimates. The ethnographic estimates tend to be orders of magnitude larger than archaeological estimates. Only the Dobe !Kung growth rate is in the range of archaeological estimates that are measured over relatively short centennial scales (Kuril Islands and Big Horn Basin in Wyoming). The archaeological estimates, on the other hand, conform more to the assumption of near stationarity than to the growth rates inferred from the ethnographic data. At first glance, this suggests that prehistoric hunter–gatherers could have been different from ethnographically documented hunter–gatherers in terms of their demography. Such a difference could stem from the nineteenth- and twentieth-century pacification and the introduction of Western medicine, which would have reduced mortality and allowed unparalleled population growth [41,42].

**Table 1.** Ethnographic and archaeological estimates of the hunter–gatherer population growth rate.

group	growth rate (%)	method	reference
ethnographic population growth rate estimates			
Dobe !Kung	0.26	intrinsic rate of population increase, which is calculated using net reproduction rate	[16] pp. 212–220
Agta	1.4	change in the census population size (1950–1965)	[17] p. 83
Asmat	1.5	change in the census population size (1956–1973)	[18] p. 457
Hadza	1.6	different methods	[9] pp. 171–174, 181, 188
Ache	2	change in the estimated population size (1930–1970)	[6] p. 83
archaeological population growth rate estimates			
Australia	0.045	approximate maximum rate 40 000–0 years ago, which is calculated from the smoothed and taphonomically corrected SPD	[13] figure 4
Australia	0.04	calculated from the temporal distribution of radiocarbon dates covering the period 5000–0 years ago	[14]
Wyoming and Colorado	0.04	calculated by fitting an exponential model to the SPD that covers the period 13 000–6000 years ago	[5]
South America	0–0.132	calculated by fitting a logistic growth model to the SPD that covers the period 14 000–6000 years ago	[4]
Kuril Islands	0.2	calculated from the smoothed SPD that covers the period 2500–2000 years ago	[11]
Wyoming (Big Horn basin)	0.31 (0.16–0.31)	calculated from the smoothed SPD; range of the mean annual growth rates during the five major growth periods; 0.31% is the maximum	[12]

However, population simulations and historical data yield similar growth rates to ethnographic data. Belovsky's simulation [30] suggests that hunter–gatherer population dynamics can easily turn into stable limit oscillations, with a wavelength between 50 and 100 years (figure 1*b*). In the simulation, the amplitude of oscillations, the long-term mean population density and the population growth rate during growth phases are controlled by environmental productivity [30]. The simulated annual population growth rates vary between 0.4% and 3%, suggesting closer affinity to the range of ethnographic rather than archaeological data.

The historical Sámi tax record data [19–21] also suggest annual population growth rates (1–3%) that are in line with ethnographic data (figure 1*c,d*). Furthermore, the Sámi data indicate similar population oscillations as Belovsky's simulation [30] (figure 1*b*). Yet, despite similar patterns, it is not clear that the population fluctuations among the Sámi are caused by similar density-dependent factors as in the simulation. Detailed historical records from the area of present-day Finland shed light on the potential causes of the population fluctuations. In the southernmost Sámi communities of the Kemi Lappi region (Kitkajärvi and Maanselkä), the population crash between 1577 and 1590 (figure 1*d*; electronic supplementary material, figure S1) is clearly related to warfare between the Russian and Swedish states [19]. In 1579, representatives of these Sámi communities complained to authorities that they had been completely ravaged by the war parties terrorizing the region [19]. However, the population decline between 1610 and 1620 and the major crash between 1645 and 1655 (figure 1*d*; electronic supplementary material, figure S1) are associated with remarks by authorities and complaints by Sámi communities about the low number of game and consequent poverty and hunger [19]. It is possible that these population declines were caused by resource depletion. It is remarkable that the major population crash between 1645 and 1655 was preceded by rapid population growth (figure 1*d*). This suggests that the population size could have overshot the regionally sustainable level with dramatic consequences. Although we cannot pinpoint the exact causes of Sámi population fluctuations, it is likely that density-dependent predator–prey dynamics played a role along with external factors such as warfare and climate.

Because the historical data and population simulation suggest population growth rate estimates that are consistent with the ethnographic data, the ethnographic estimates can hardly be the result of recent pacification or medical aid. Instead, they seem to reflect more permanent characteristics of hunter–gatherer demography. Then the question becomes: why are ethnographic and archaeological population growth-rate estimates so different? The simple answer could be that the resolution of archaeological population proxies is not sufficiently high to detect such fine-grained dynamics as historical data and simulations suggest, and therefore, proxies fail to capture true growth rates.

**Figure 2.**
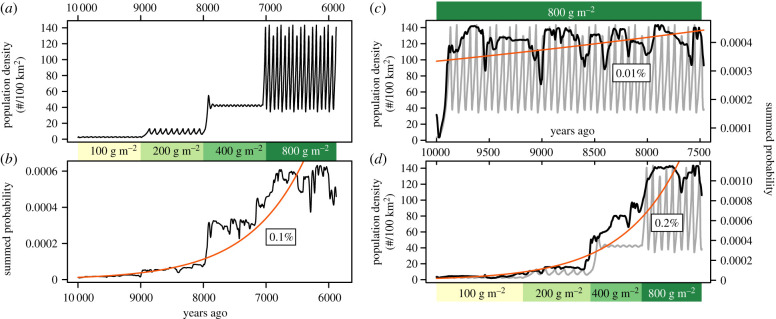
Simulated archaeological proxies of hunter–gatherer population dynamics. (*a*) Simulated hunter–gatherer population trajectory based on Belovsky's simulation [30]. Different regimes of environmental productivity are shown below the curve. These regimes correspond to different long-term mean population densities. (*b*) Archaeological proxy (SPD) of the population pattern shown in (*a*). The proxy is based on dates sampled under the assumption that their distribution follows the underlying pattern shown in (*a*). (*c,d*) Underlying population patterns (grey curves) and their archaeological proxies (black curves) in environments with constant (*c*) and changing (*d*) productivity. Productivity regimes are shown above (*c*) and below (*d*) curves. (*b–d*) Show exponential growth models and annual growth rates (%).

To test this idea, we created SPDs that are based on simulated data assuming that past hunter–gatherer population dynamics are characterized by high-frequency fluctuations, as suggested by simulations and historical data. Figure 2*a,b* suggests that an SPD is not able to capture such high-frequency fluctuations and instead only represents an approximation of the mean trend of the underlying actual dynamics. This inability is independent of simulated sample (electronic supplementary material, figure S2) and is most likely an inherent limitation of the SPD method. There are, nevertheless, two instances when the SPD seems to correctly track short-term features of the underlying simulated pattern, namely the regime shifts in environmental productivity and, consequently, in population dynamics 9000 and 8000 years ago (figure 2*a,b*). However, this is possible because of abrupt step-like productivity changes in the simulated pattern, while more gradual changes in productivity and population dynamics regimes have, most likely, been more common in reality.

Despite the inability to detect high-frequency variation in population size, the SPD captures changes in the long-term mean population density that are determined by the environmental productivity in the population dynamics simulation [30]. The long-term annual population growth rate (0.1%) measured over the entire time range is close to the long-term annual rate of change in environmental productivity (0.07%) (figure 2*a,b*).

These results are confirmed by wavelet coherence analyses (electronic supplementary material, figure S3). They show significant similarities between the simulated pattern and SPDs at the time-scale of around 1000 years, i.e. at the scale of regime shifts in productivity and population dynamics. The wavelet coherence analyses also highlight significant similarities around 8000 years ago and, in some of the samples, 9000 years ago (electronic supplementary material, figure S3). However, besides these two instances, the wavelet coherence analyses do not show significant similarities at the period length of 50 or 100 years, which are the wavelengths of the oscillations in the simulated pattern. Thus, our results suggest that it is not usually possible to detect the underlying ’true’ high-frequency fluctuations in population size using SPDs. Previously, it has been shown that slightly longer duration population events of 100–200 years can, however, be captured by SPDs [43].

Measuring high-frequency fluctuations from SPDs would often produce spurious results because of the noise created by calibration and sampling variation [34]. This noise is evident among the sample of SPDs, where most of them show features related to calibration, such as amplified peaks and troughs (electronic supplementary material, figure S2). Most likely, archaeologists are aware of this problem, as population growth rates are usually estimated over longer time periods.

Our results suggest that growth rates measured over several millennia would reflect changes in long-term mean population size or density and simultaneously in the rate of change in environmental productivity. However, this measure would miss the underlying actual population dynamics. This is highlighted by two simulated population trajectories and their archaeological proxies (figure 2*c,d*). Figure 2*c* shows long-term population dynamics under conditions of high and stable environmental productivity, whereas in figure 2*d*, productivity increases through time. Population growth rates measured from the respective SPDs suggest that the population in figure 2*c* is almost stationary while the population in figure 2*d* is more dynamic with a much higher growth rate. Yet, it is clear that the underlying population dynamics in figure 2*c* are far from stationary, but because the long-term mean population density does not change, the resulting SPD suggests a very low growth rate. In figure 2*d*, the underlying dynamics mostly contain less dramatic high-frequency fluctuations, but because the long-term mean population density changes as a result of productivity changes, the resulting SPD indicates changing population size.

## 4. Discussion and conclusion

Our results demonstrate that archaeological growth-rate estimates of prehistoric hunter–gatherers are different from hunter–gatherer population growth rates derived from ethnographic, historical or simulated data. Simultaneously, our results suggest that this difference arises from the inability of archaeological population proxies to detect changes in the actual population size that occur over short periods of time. We argue that growth rates measured from archaeological population proxies over long periods (centennial to millennial scale) reflect changes in the long-term mean population size or density and, most likely, miss the actual underlying high-frequency variation. Thus, it seems that ethnographic and historical estimates of hunter–gatherer population growth rates measure different things, and therefore, archaeological and ethnographic estimates are not directly comparable. This implies that the difference between archaeological and ethnographic estimates does not represent demographic differences between prehistoric and more recent hunter–gatherers of the ethnographic record. We do not dispute the possibility that past and recent hunter–gatherers might have been demographically different, but the argument cannot be based on growth rate estimates.

The long-term mean population density in the simulated population pattern is controlled by environmental productivity, which is independent of the hunter–gatherer population size [30]. This finds support in the ethnographic data, showing that environmental productivity exerts a strong influence on hunter–gatherer population density [44–46]. Most likely, this holds for prehistoric hunter–gatherers as well. Analyses of the relationship between archaeological population proxies and proxies of environmental productivity that are geographically well-linked to archaeological data have demonstrated that periods of population growth and decline were related to increases and decreases of environmental productivity [12,32,33]. We argue that in the case of prehistoric hunter–gatherers, archaeological SPDs measure long-term mean population size, which, in turn, tracks environmental productivity. Consequently, the long-term mean population size reflected in SPDs can be considered as the carrying capacity, i.e. theoretical equilibrium population size, around which the true population size fluctuates. However, the true population size is rarely pinpointed by proxies because of the coarse resolution of the archaeological data.

Ethnographic and historical records yield population growth rates that are directly linked to births and deaths and in and out migrations in the population. However, because of the difference in temporal resolution and scale, we argue that this link is much vaguer in the growth rates measured from archaeological proxies. Therefore, one has to be cautious when applying population ecology models to archaeological reconstructions of population dynamics [4]. Long-term patterns in archaeological proxies may resemble, for example, logistic growth of saturating population size even though the pattern is actually a result of changes in the environmentally driven carrying capacity, where carrying capacity first increases and then stabilizes. Actual changes in the population size that follow a logistic growth model occur within time intervals that are usually beyond the resolution of archaeological proxies. Caution is equally required when inferring detailed demographic parameters, such as total fertility of prehistoric hunter–gatherer women, from demographic models parametrized using long-term growth rates measured from archaeological proxies [5]. Such models may yield meaningless results if the growth rate is not closely related to births and deaths in the study population.

Our results lead also to a potential explanation of the forager population paradox, i.e. the contradiction between the high growth rates of recent hunter–gatherers and the stationarity of the Palaeolithic and Mesolithic hunter–gatherers [9]. The ostensible stationarity results from the fact that, as long as humans are hunter–gatherers, their population size is bound by nature. The population size of hunter–gatherers, who rely on the productivity of natural resources, cannot grow above the level sustained by regional productivity for extended periods. Therefore, we should not expect to see long-term hunter–gatherer population growth that would be independent of a corresponding increase in environmental productivity. Similarly, if environmental productivity declines, the hunter–gatherer population would decline accordingly. This explains why we can have both annual population growth rates of up to 3% and still relatively small hunter–gatherer populations: rapid growth periods occur on a relatively short-term basis, whereas long-term growth is limited by environmental carrying capacity.

**Figure 3.**
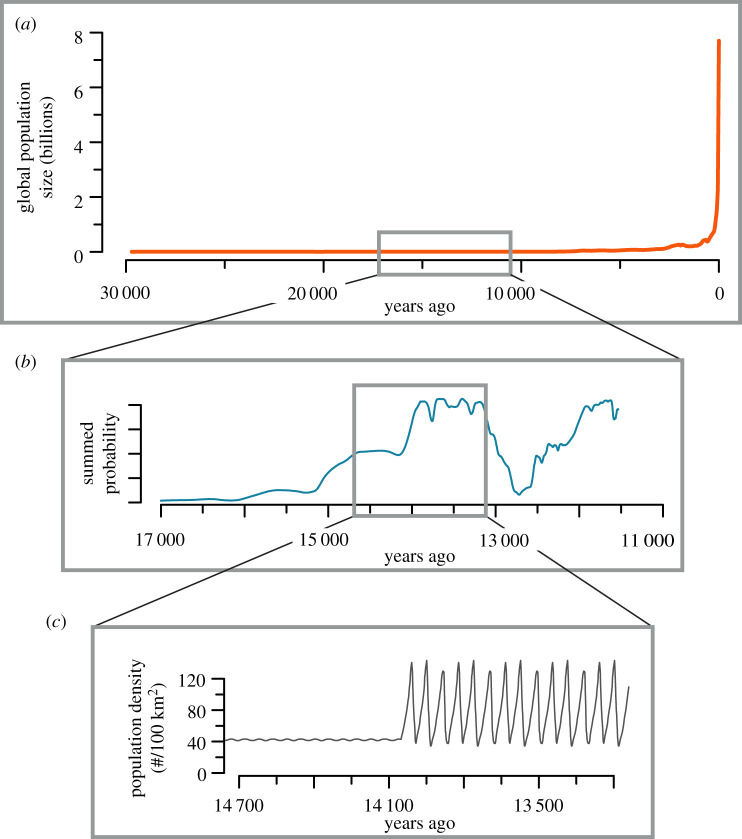
Schematic of different scales of analyses and the population dynamics of hunter–gatherers. (*a*) Global-scale trajectory of human population size [47] implies that hunter–gatherer populations have been stationary. Prehistoric sections of such reconstructions are usually assumed rather than inferred from data. (*b*) Continental-to-regional scale, which is trackable with archaeological proxies, suggests that long-term population dynamics are characterized by periods of growth and decline. (*c*) The scale of actual population size is maybe the most dynamic, but it is usually beyond the resolution of archaeological methods. (Online version in colour.)

Thus, the stationarity of prehistoric hunter–gatherer populations in the global-scale human population growth reconstructions [47] is, of course, more apparent than real (figure 3). Stationarity is apparent when compared to the astronomical population growth of the historical era, but in any particular area, stationarity of hunter–gatherer populations is hardly a valid assumption (figure 3*a,b*). As one shifts the focus to continental or regional scales, archaeological proxies demonstrate fluctuations in the long-term mean population size (figure 3*b*). If one would be able to further sharpen the focus, it would enable us to observe the actual trajectories of prehistoric hunter–gatherer population sizes (figure 3*c*). This last scale, we believe, currently eludes archaeologists (and possibly always will) because of the temporal resolution of archaeological data.

Although we have been critical of the estimation of population growth rates from archaeological proxies, this is not aimed at critiquing archaeological reconstructions of prehistoric population dynamics in general or SPDs in particular. One should not read into this paper that SPDs are worthless. We strongly believe that the method using temporal distributions of archaeological radiocarbon dates captures real signals of long-term human population dynamics. What we want to highlight, however, is that to make a lasting contribution to human demography and population ecology, archaeologists need to acknowledge the differences in scale and resolution between archaeological data and the data that demographers and ecologists usually analyse. In addition, archaeologists need to acknowledge the potential consequences these differences may have for the applicability of demographic and ecological models in archaeology.

## Supplementary Material

Supplementary figures S1-S3

## Data accessibility

All data and scripts have been deposited to the Zenodo data repository: http://doi.org/10.5281/zenodo.3734019.
