# Supplementary material for: Why are population growth rate estimates of past and present hunter–gatherers so different?
Source: Philos Trans R Soc Lond B Biol Sci. 2020 Nov 30;376(1816):20190708. doi: 10.1098/rstb.2019.0708 (PMC7741106; doi:10.1098/rstb.2019.0708)
Supplement: Supplementary figures S1-S3 [file rstb20190708supp1.pdf]

Philosophical Transactions B

Why are population growth rate estimates of past and present hunter-gatherers so different?

Supplementary material

Miikka Tallavaara[1*] and Erlend Kirkeng Jørgensen[2]

[1]Department of Geosciences and Geography, University of Helsinki, P.O. Box 64, 00014 University of Helsinki, Finland
ORCID ID: https://orcid.org/0000-0001-9252-7309
[2]UiT - The Arctic University of Norway. Department of Archaeology, History, Religious Studies and Theology. Postbox 6050 Langnes. 9037 Tromsø.
ORCID ID: https://orcid.org/0000-0002-8573-4489

*Author for correspondence (miikka.tallavaara@helsinki.fi).

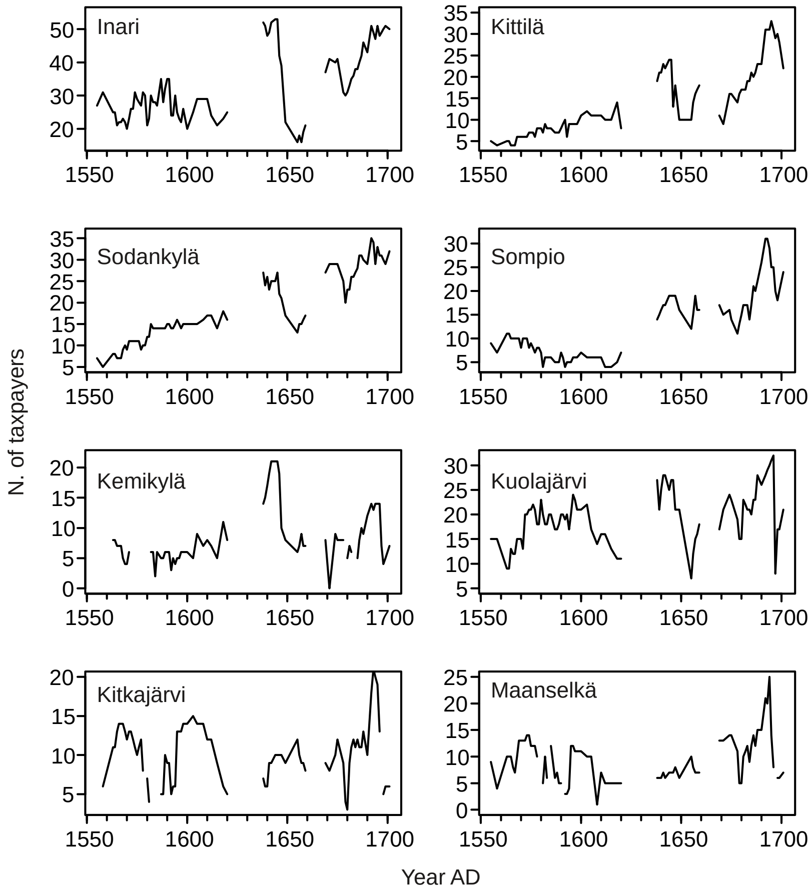

Figure S1. Number of taxpayers in individual Sámi communities in the historical Kemi Lappi region in northern Finland [1,2].

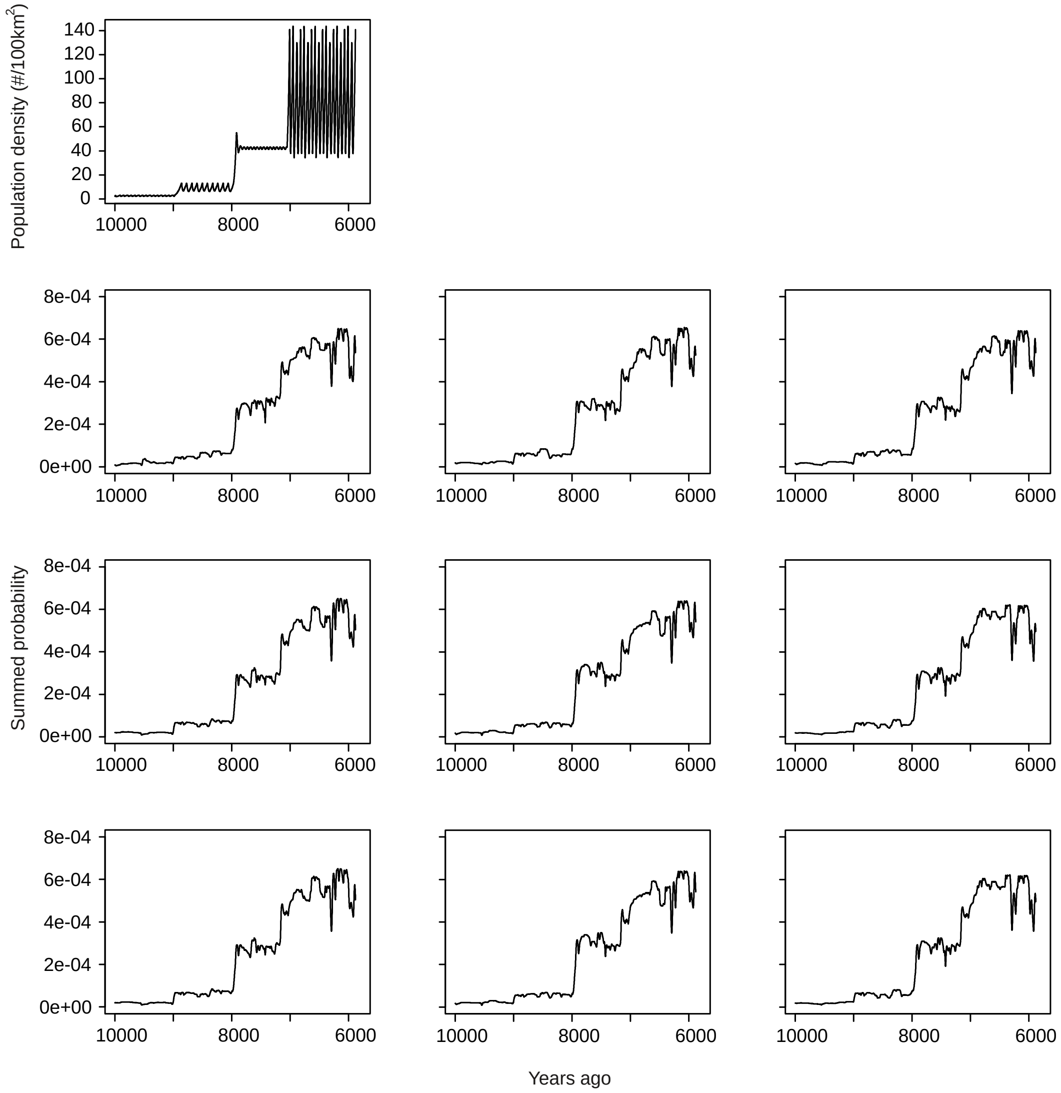

Figure S2. SPDs based on nine different random samples (N=5000) of calendar dates from the underlying population pattern shown on the top row of the figure. The underlying population pattern is based on simulation [3]

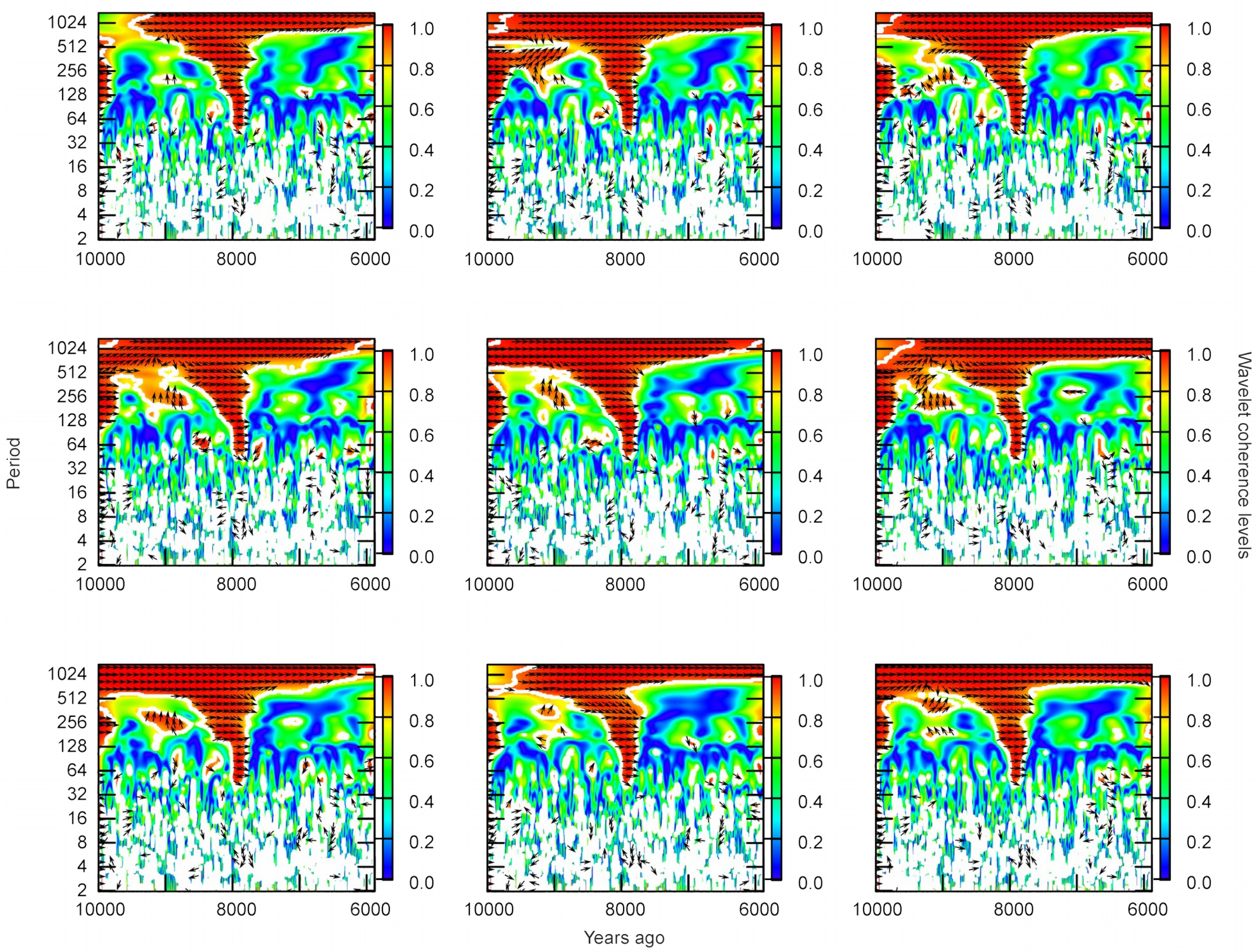

Figure S3. Wavelet coherence plots showing the similarity between sample SPDs and the underlying actual population pattern. Significant similarities in the periodicity are indicated by the white contour and red colour. Horizontal arrows pointing to the right indicate that the two series (actual pattern and SPD) are in phase at the respective period. Arrows pointing to the left indicate that the two series are in anti-phase.

References

1. Vahtola A. 2003 *Kemin Lapin saamelaisten verotus ja alueen saamelainen asutus 1555-1673 (Unpublished Licentiate Thesis)*. Department of History, University of Oulu.

2. Enbuske M. 2008 *Vanhan Lapin valtamailla: asutus ja maankäyttö historiallisen Kemin Lapin ja Enontekiön alueella 1500-luvulta 1900-luvun alkuun*. Helsinki: Suomalaisen Kirjallisuuden Seura.

3. Belovsky GE. 1988 An optimal foraging-based model of hunter-gatherer population dynamics. *J. Anthropol. Archaeol.* **7**, 329–372. (doi:10.1016/0278-4165(88)90002-5)
